# Vascular endothelial cells facilitated HCC invasion and metastasis through the Akt and NF-κB pathways induced by paracrine cytokines

**DOI:** 10.1186/1756-9966-32-51

**Published:** 2013-08-13

**Authors:** Yao-Hui Wang, Yin-Ying Dong, Wei-Min Wang, Xiao-Ying Xie, Zhi-Ming Wang, Rong-Xin Chen, Jie Chen, Dong-Mei Gao, Jie-Feng Cui, Zheng-Gang Ren

**Affiliations:** 1 Liver Cancer Institute, Zhongshan Hospital, Fudan University and Key Laboratory of Carcinogenesis and Cancer Invasion, Ministry of Education, 136 Yi Xue Yuan Road, 200032, Shanghai, PR China; 2 Department of Radiology, Shanghai Cancer Center, Fudan University, 200032, Shanghai, PR China; 3 Department of Oncology, Zhongshan Hospital Subdivision, Fudan University, 200052, Shanghai, PR China

**Keywords:** Hepatocellular carcinoma, Endothelial cell, Cytokine, Invasiveness, Metastasis

## Abstract

**Background:**

It is well documented that cancer cells secrete angiogenic factors to recruit and sustain tumor vascular networks. However, little is known about the effects of endothelial cells on the behavior of tumor cells. The study here was to determine the roles of endothelial cells in HCC cell growth, migration and invasion.

**Methods:**

A mixture of highly metastatic MHCC97H cells and HUVEC cells, as well as MHCC97H cells alone were subcutaneously injected into nude mice to observe the effects of HUVECs on HCC growth. The biological characteristics of MHCC97H cells respectively treated with conditioned medium (CM) derived from HUVECs and endothelial cell basal medium (EBM) in vitro, such as proliferation, migration and invasion, invasion/metastasis associated gene expression, were comparatively analyzed. Differential cytokines between CM and EBM were screened and identified using human cytokine array. Effects of the interested differential cytokine CCL2, IL-8 and CXCL16 and its related signaling pathways were further investigated in HCC cells.

**Results:**

Subcutaneous tumorigenicity of MHCC97H cells in nude mice was promoted by HUVECs and its invasion/metastasis associated genes were significantly upregulated. The in vitro, proliferation, migration and invasion of HCC cells treated with CM were all significantly enhanced as compared to those with EBM stimulation. Simultaneously, PI3K/Akt and ERK1/2 pathway in HCC cells were activated by CM. Total of 25 differential cytokines were identified between CM and EBM such as angiopoietin-2, CCL2 (MCP-1), uPA, endostatin, CXCL16, IL-8, pentraxin 3 etc. The selected differential cytokines CCL2, IL-8 and CXCL16 all modulated the expressions of HCC invasion/metastasis genes, especially MMP2 and MMP9. In exposure to CCL2 or CXCL16 alone, upregulation in AKT phosphorylation but no change in ERK phosphorylation were found in MHCC97H cells, moreover the contents of nuclear transcription factor NF-κB were increased as compared to the control. However, no effects on the activation of Akt and ERK pathway in MHCC97H were found in exposure to IL-8.

**Conclusion:**

This study expands the contribution of endothelial cells to the progression of HCC. It unveils a new paradigm in which endothelial cells function as initiators of molecular crosstalks that enhance survival, migration and invasion of HCC cells.

## Introduction

Hepatocellular carcinoma (HCC) is the fifth most common cancer worldwide and the third leading cause of cancer-related death [1]. Although significant advances in surgical techniques and perioperative care over the last two decades, the long-term prognosis of HCC remains dismal largely due to the high frequency of metastasis or recurrence. Recently, more evidences suggest that HCC metastasis involves a complex cascade of signal events between tumor cells and host stroma microenvironment. These crosstalking might modulate or determine the process of HCC invasion and metastasis. Thus, exclusive reliance on tumor cell itself for research cannot enable insight into the diverse pathological changes occurring in HCC metastasis. Generally, the microenvironment of HCC is composed of stromal cells (e.g., hepatic stellate cells, fibroblasts, invading inflammatory/immune cells, and endothelial cells) and non-cellular components (e.g., growth factors, proteolytic enzymes, inflammatory cytokines, and extensive extracellular matrix proteins). A lot of studies on HCC have validated the important roles of stromal cells in HCC progression [2]. Hepatic stellate cells (HSCs) increase HCC growth and invasion both in vitro and in vivo. Conditioned media derived from HSCs induce HCC cell proliferation and migration. Moreover, on a three-dimensional spheroid co-culture system as well as an in vivo implantation of a mixture of HSCs and HCC cells, HSCs obviously accelerate HCC growth and diminish the extent of central necrosis [3,4]. Activated HSCs also enhance HCC progression by other means such as regulating T cells that create an immunosuppressive microenvironment and stimulating angiogenesis [5]. Through the release of different factors like cytokines, chemokines, or enzymes, tumor-associated macrophages (TAMs) can regulate tumor growth, angiogenesis, invasion, and metastasis [6]. Particularly, some secreted factors from TAMs also induce cancer cell motility, thereby enhancing tumor cell invasion capacity [7]. These data demonstrate that stromal cells can actively modulate the malignant characteristics of HCC cells and further determine the outcome of HCC.

Given that tumors have abundant blood vessels for supplying oxygen and nutrition, endothelial cells (ECs) are ubiquitous within solid tumors. In other solid tumors, ECs modulate various pathophysiological processes [8,9]; in HCC, ECs directly influence cancer progression through neoangiogenesis [10]. However, the molecular framework of this crosstalk in the context of a specific tissue and its consequences on HCC metastasis are largely unknown. Thus, the counteractive effects of ECs on HCC cell behaviors in cancer development and progression merit to be investigated. In this study, we provided some evidences that EC-initiated signaling directly affected the malignant progression of HCC cells in vitro and in vivo, and that the induction of PI3K/Akt and NF-κB activation may be responsible for these effects.

## Materials and methods

### Cell lines and animals

The MHCC97H cells (established in the Liver Cancer Institute of Fudan University [11]) were cultured in Dulbecco’s modified Eagle’s medium (DMEM, Gibco) supplemented with 10% fetal bovine serum (FBS, Gibco). Human Umbilical Vein Endothelial Cells (HUVECs, ScienCell) were cultured in EC basal medium (EBM; ScienCell) with additional 10% FBS, and guaranteed to subcultured for three population doublings. Male BALB/c nu/nu mice (3-4 week old; SLAC Laboratory Animal Co, Ltd, Shanghai, China) were housed in specific pathogen-free conditions. All animal protocols were approved by the Ethical Committee on Animal Experiments of the University of Fudan Animal Care Committee, Shanghai, China (Permit Number: SYXK:2008-0039). All efforts were made to minimize suffering.

### Collection of conditioned medium (CM) from HUVECs

After HUVEC growth in a T75 flask reached approximately 80% confluency, the medium was changed with complete endothelial cell basal medium (EBM) containing 10% FBS (20 mL/T75) and incubated for 24 h. The same medium was incubated for 24 h in a T75 flask without HUVECs to serve as a control. The collected supernatant was concentrated by a centrifugal filter (Millipore, Schwalbach, Germany) at 4000 rpm for 30 min at 4°C. The concentrated supernatant was then filtered through 0.2 μm filters and stored at −80°C for further use. The protein concentration of the concentrated supernatant was measured by BCA protein assay (Pierce).

### Subcutaneous tumorigenicity test of MHCC97H cells premixed with HUVECs

Nude mice were subcutaneously injected at the upper left flank region with 0.1 mL of cell suspension containing either 5 × 10^6^ MHCC97H cells or a mixture of 5 × 10^6^ MHCC97H cells and 1 × 10^6^ HUVECs. Tumor growth was evaluated by measuring the length and width of tumor mass at the inoculation site. After 10 days, the tumor-bearing mice were sacrificed. The tumors were removed and fixed in 4% formaldehyde for pathological analysis and snap frozen in liquid nitrogen for gene expression analysis.

### Cell proliferation assay

About 100 μl of MHCC97H cells (6 × 10^3^ cells) with DMEM containing 10% FBS were seeded into a 96-well plate. At the indicated time points, 10 μl of CCK-8 solution (Dojindo, Japan) was added to the cells and incubated for 1 h. The number of viable cells in each well was tested by color absorbance at 450 nm.

### Wound healing assay, cell invasion assay, and cell motility assay

Scratch wound healing assay was performed to assess cell migration. In brief, 3 × 10^4^ MHCC97H cells were cultured in a 24-well plate for 24 h. After a tight cell monolayer was formed, the cells were incubated with serum-free medium for 24 h and the cell monolayer was wounded with a plastic pipette tip. The remaining cells were washed twice with fresh medium to remove cell debris, and further incubated with CM or EBM for 24 and 48 h. At the indicated time points, the migrant cells at the wound front were photographed with a microscope.

The cell invasive assay was the same as in our previous study with minor modifications [12]. Briefly, 1 × 10^5^ MHCC97H cells in 100 μl of serum-free DMEM were placed into the upper compartment of a boyden chamber (Costar) precoated with Matrigel, and 600 μl defined medium containing CM or EBM was added to the lower compartment as a chemoattractant. After incubating for 48 h, the cells that failed to penetrate the filters were gently removed by cotton swabs. The invading cells in the membrane were fixed with 4% formaldehyde in PBS (Gibco), stained in Giemsa for 10 min, and then counted under a light microscope. Cell motility assay was performed similarly except that an uncoated filter was used and the incubation time was 18 h.

### Quantitative reverse transcription polymerase chain reaction (qRT-PCR)

Total RNA from cells was extracted using Trizol reagent (Invitrogen, Karlsruhe, Germany) according to the manufacturer’s protocol. The complementary DNA (cDNA) was synthesized using the Superscript First-Strand Synthesis System (Thermo Scientific, Epsom, UK) and used as template for RT-PCR with a gene specific primer and SYBR Green PCR Master Mix kit (Invitrogen, Karlsruhe, Germany). Relative gene expression was normalized to GAPDH and reported as 2^-ΔCt^ [ΔCt = Ct (MMP2 or other gene)-Ct (GAPDH)]. The primer sequences of matrix metalloproteinase 2 (MMP2), MMP9, CD44, and osteopontin (OPN) are listed in Table 1.

**Table 1:** Primer pairs used for qRT-PCR

**Gene symbol**	**Sequence 5′-3′**
MMP2	FORWARD:5′-GTTCATTTGGCGGACTGT-3′
	REVERSE:5′-AGGGTGCTGGCTGAGTAG-3′
MMP9	FORWARD:5′-CTTTGGACACGCACGAC-3′
	REVERSE:5′-CCACCTGGTTCAACTCACT-3′
CD44	FORWARD:5′-GGTGAACAAGGAGTCGTC-3′
	REVERSE:5′-TTCCAAGATAATGGTGTAGGTG-3
SPP1	FORWARD:5′-CAGTGATTTGCTTTTGCC-3′
	REVERSE:5′-AGATGGGTCAGGGTTTAG-3′
GAPDH	FORWARD:5′-CTCCTCCACCTTTGACGC-3′
	REVERSE:5′-CCACCACCCTGTTGCTGT-3′

*qRT-PCR* quantitative real time reverse transcription polymerase chain reaction, *F* forward, *R* reverse.

### Western blot analysis

Protein extraction and Western blot analysis were performed as in our previous work [13]. Primary antibodies were diluted with TBSA as follows: p-Akt (Ser473, 1:1000; Cell Signaling Technology, Boston, USA), Akt (1:1000; Cell Signaling Technology, Boston, USA), p-ERK (Thr202/Tyr204, 1:1000; Cell Signaling Technology, Boston, USA), ERK (1:1000; Cell Signaling Technology, Boston, USA), and GAPDH (1:1000; Kangchen). Secondary antibodies were diluted with TBSA (against mouse and rabbit, 1:5000; Dingguo Bio, Beijing, China).

### Immunohistochemistry and immunocytochemical assays

Immunohistochemical staining was performed based on the method of Tang [14]. In a typical procedure, after rehydration and antigen retrieval, cell slides were incubated with diluted primary antibody against human p-Akt (1:50; Cell Signaling Technology, Boston, USA) and p-ERK (1:50; Cell Signaling Technology, Boston, USA) at 4°C overnight, followed by the secondary antibody conjugated with HRP (anti rabbit, 1:200; Dingguo Bio Beijing, China) at 37°C for 30 min. Staining was carried out with 3,3′-diaminobenzidine (DAB) and counter-staining was conducted with Mayer’s hematoxylin. Cell immunocytochemical assay was performed similar to the above method except for the cell coverslip preparation and fixation, as well as the use of primary antibodies against Ki67 (1:100; Dako, Copenhagen, Denmark), MMP2 (1:100; Santa Cruz Biotechnology, Heidelberg, Germany), and MMP9 (1:100; Cell Signal Technology, Boston, USA).

### Human cytokine array

Angiogenesis-related protein expression in CM and EBM was evaluated by a semiquantitative technique (Proteome Profiler™, Human Angiogenesis Array Kit, R&D Systems, Minneapolis, USA) according to the manufacturer’s instructions. The selected capture antibodies were spotted in duplicate on nitrocellulose membranes. Samples were diluted and mixed with a cocktail of biotinylated detection antibodies. The sample/antibody mixture was then incubated with a Human Angiogenesis Array kit. Any protein/detection antibody complex present was bound by its cognate-immobilized capture antibody on the membrane. After washing to remove unbound materials, streptavidin-HRP and chemiluminescent detection reagents were sequentially added. Light was produced at each spot in proportion to the amount of bound analyte. Data were captured by exposure to X-ray films. Array signals from the scanned X-ray film images were analyzed using Image J. The results were expressed as fold changes above or below the unexposed cultures.

### Evaluation of nuclear factor-κB (NF-κB) DNA binding activity

The nuclear extracts and DNA-binding activity of NF-κB in MHCC97H cells were prepared according to the instruction of Active Motif. Briefly, after treating HCC cells with cytokine CCL2 (chemokine C-C motif ligand 2, R&D Systems, Minneapolis, USA), IL-8 (interleukin-8, Sigma, Tokyo, Japan), and CXCL16 (chemokine C-X-C motif ligand 16, R&D Systems, Minneapolis, USA) for 24 h, MHCC97H cells were collected in ice-cold PBS with phosphate inhibitors and centrifuged at 500 rpm for 5 min. The pellets were resuspended and treated with a detergent. After removing the cytoplasmic fraction by centrifugation at 14 000 × *g* for 30 s, nuclei were harvested and lysed in lysis buffer with the protease inhibitor cocktail for nuclear protein extraction.

The content of NF-κB binding to DNA in nuclear extracts was measured using specific TransAM NF-κB p65 assay (active motif). A 96-well plate was precoated with an oligonucleotide containing the NF-κB p65 binding consensus site. The active form of the p65 subunit was detected using antibodies specific for an epitope that was accessible only when the appropriate subunit bound to its target DNA. An HRP-conjugated secondary antibody provided a colorimetric readout that was quantified by a spectrophotometer (450 nm).

### Statistical analysis

Data were analyzed using SPSS software (version 16.0). Results were expressed as the mean ± SD. Statistical analysis was performed by one-way ANOVA and Student’s *t*-test. *P* < 0.05 was considered statistically significant.

## Results

### Effects of HUVECs on the tumorigenicity of MHCC97H cells in vivo

**Figure 1:**
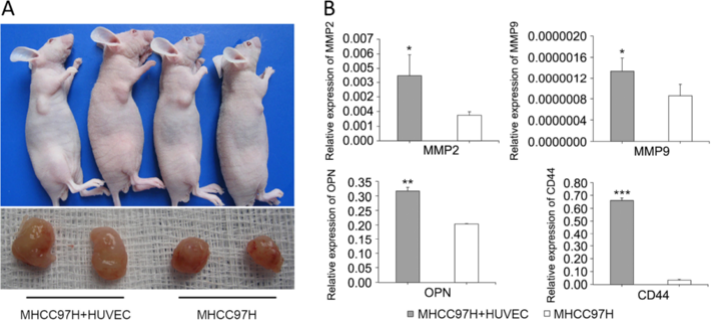
**Subcutaneous tumorigenicity test of MHCC97H cells premixed with HUVECs and the expression of HCC invasion/metastasis-associated genes. (A)** MHCC97H cells as well as a mixture of MHCC97H cells and HUVECs were subcutaneously implanted into nude mice as described in the “Material and methods” section. Representative tumors resected from nude mice appeared 10 days after implantation. **(B)** The expression of MMP2, MMP9, OPN, and CD44 were detected by qRT-PCR in subcutaneous tumors (*P < 0.05, **P < 0.01, ***P < 0.001 vs. MHCC97H cells alone).

To assess the effects of HUVECs on the tumorigenicity of HCC cells, we injected subcutaneously MHCC97H cells into nude mice either alone or in combination with HUVECs. Subcutaneous tumors developed at the site of implantation in mice. The tumor size in mice implanted with a mixture of HUVECs and MHCC97H cells were much larger than that in mice implanted with MHCC97H cells alone (Figure 1A). In addition, the expression of HCC invasion/metastasis-associated genes (MMP2, MMP9, OPN, and CD44) in the subcutaneous mixed tumor of MHCC97H cells and HUVECs were significantly higher than those formed by MHCC97H cells alone (*p < 0.05; Figure 1B).

### Changes in the malignant properties of HCC cells under CM stimulation

**Figure 2:**
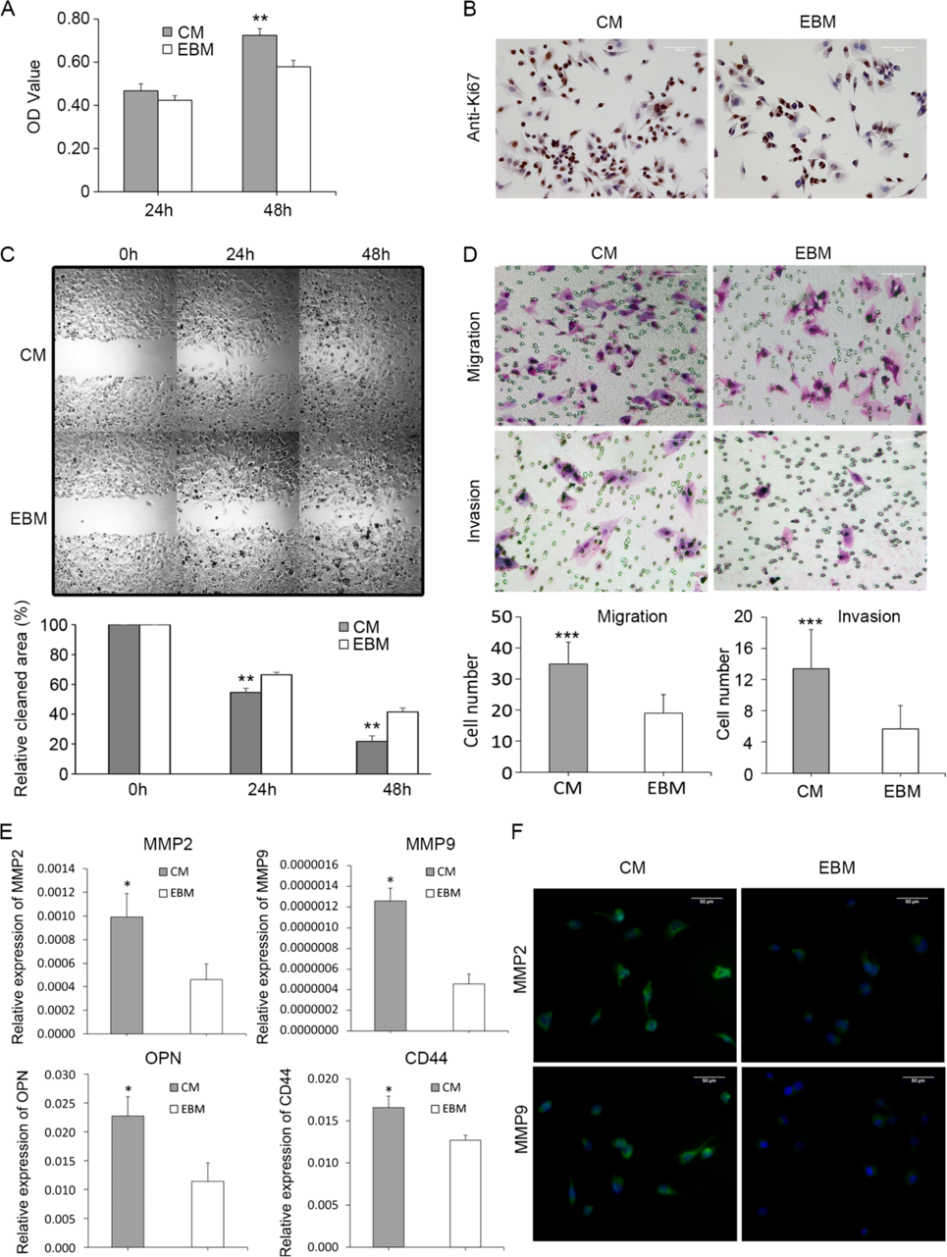
**Changes in the malignant properties of HCC cells under CM stimulation. (A)** CM significantly promoted HCC cell proliferation (**P < 0.01 vs. EBM at 48 h) was measured by CCK8. **(B)** The expression of Ki67 in the nucleus of HCC cells. **(C)** Wound healing assays were performed with MHCC97H cells incubated by CM or EBM. The amount of migrating cells at the wound front was much higher than that in the control (**P < 0.01). **(D)** In the cell motility/invasion assay, the average numbers of penetrating HCC cells induced by CM were all obviously higher than those induced by EBM (***P < 0.001). **(E)** CM significantly increased the expression of HCC invasion/metastasis-associated genes in HCC cells compared with EBM (*P < 0.05). **(F)** High expression of MMP9 and MMP2 were confirmed in MHCC97H cells by immunofluorescent staining.

As shown in Figure 2A and B, the proliferation of HCC cells treated with CM derived from HUVECs significantly increased compared with that treated with EBM (*p < 0.05). The numbers of nuclear Ki67-positive cells in the MHCC97H cells treated with CM also increased. These results supported that some secreted factors derived from HUVECs may stimulate HCC cell proliferation in vitro.

Wound healing assay revealed that the amount of migrating cells at the wound front were much higher than that of the control (Figure 2C). It suggested that the migratory capability of HCC cells can be significantly enhanced by CM from HUVECs. Cell motility assay demonstrated that under induction by CM, the average number of MHCC97H cells (34.9 ± 2.3) that penetrated the filters increased compared with induction by EBM (19.0 ± 3.6; Figure 2D).

The numbers of invading MHCC97H cells induced by CM (13.4 ± 1.5) were obviously higher than those induced by EBM (5.7 ± 1.2) in cell invasion assay. (Figure 2D). On the other hand, the expression of MMP2, MMP9, OPN, and CD44 were also remarkably upregulated in MHCC97H cells treated with CM compared with those treated with EBM (Figure 2E). Moreover, high expression of MMP2 and MMP9 was confirmed using immunofluorescent staining (Figure 2F). Combined with the aforementioned results of cell migration, the distinct increase in cell invasion ability under CM stimulation can be associated with the enhanced cell motility and upregulation of MMPs.

### CM induced the activation of the PI3K/Akt and ERK pathways in HCC cells

**Figure 3:**
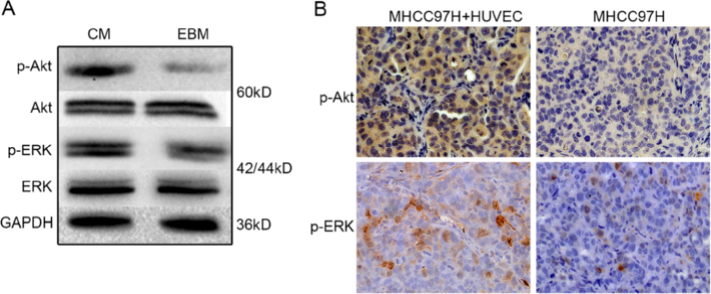
**Effects of CM on PI3K/Akt and ERK pathway activation in HCC cells. (A)** Expression of p-Akt and p-ERK in MHCC97H cells under CM or EBM stimulation were detected by Western blot. **(B)** Expression of p-Akt and p-ERK in subcutaneous tumors derived from a mixture of MHCC97H cells and HUVECs were analyzed by immunohistochemistry.

Activation of the PI3K/Akt and ERK pathways by CM is reportedly involved in regulating the invasion and metastasis in HCC cells [15]. In the present study, the levels of Akt and ERK phosphorylation in MHCC97H cells under CM stimulation were elevated compared with that in the control cells (Figure 3A). High expression of phosphorylated Akt and phosphorylated ERK was also found in subcutaneous tumor formed by MHCC97H cells premixed with HUVECs compared with that formed by MHCC97H cells alone (Figure 3B). These data verified that CM induced the activation of the PI3K/Akt and ERK pathways in HCC cells.

### Screening of the content of differential cytokines between CM and EBM

**Figure 4:**
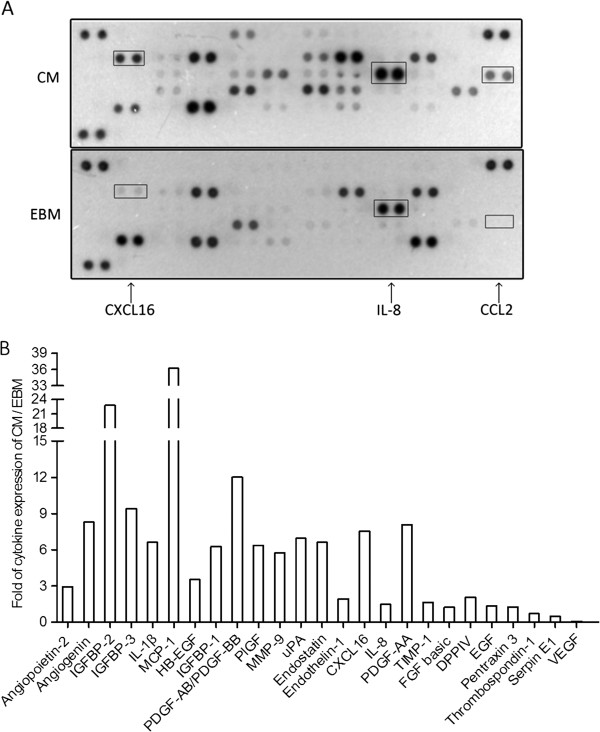
**Profile analysis of differentially expressed cytokines. (A)** Screening of different cytokines using a human cytokine array between CM (top panel) and EBM (bottom panel). **(B)** A total of 25 differentially expressed cytokines were expressed as fold changes above or below the control.

A human cytokine array (Figure 4A) comprising 55 different cytokines was used to screen the content of differential stimulatory factors between CM and EBM. A total of 25 differential cytokines were found in CM (Figure 4B and Table 2). Among them, 22 were upregulated [angiopoietin-2, angiogenin, IGFBP-2, IGFBP-3, CCL2 (also known as monocyte chemoattractant protein-1, MCP-1), IGFBP-1, MMP-9, uPA, endostatin, CXCL16, endothelin-1, IL-8, TIMP-1, etc.] and 3 were downregulated (pentraxin 3, serpin E1, and VEGF). Some factors among the identified differential factors may be involved in the regulation of HCC cell growth, migration, and invasion as the results mentioned above.

**Table 2:** Relative expression of CM/EBM

**Angiogenesis factors**	**Fold (CM/EBM)**	**Angiogenesis factors**	**Fold (CM/EBM)**
Angiopoietin-2	2.94	Endothelin-1	1.95
Angiogenin	8.29	CXCL16	7.54
IGFBP-2	22.78	IL-8	1.48
IGFBP-3	9.41	PDGF-AA	8.09
IL-1β	6.62	TIMP-1	1.63
MCP-1	36.24	FGF basic	1.24
HB-EGF	3.51	DPPIV	2.08
IGFBP-1	6.25	EGF	1.36
PDGF-AB/PDGF-BB	12.01	Pentraxin 3 (PTX3)	1.27
PlGF	6.36	Thrombospondin-1	0.72
MMP-9	5.74	Serpin E1	0.48
uPA	6.97	VEGF	0.08
Endostatin/Collagen XVIII	6.64		

### CCL2, IL-8, and CXCL16 regulated the expression of invasion- and metastasis-associated genes

**Figure 5:**
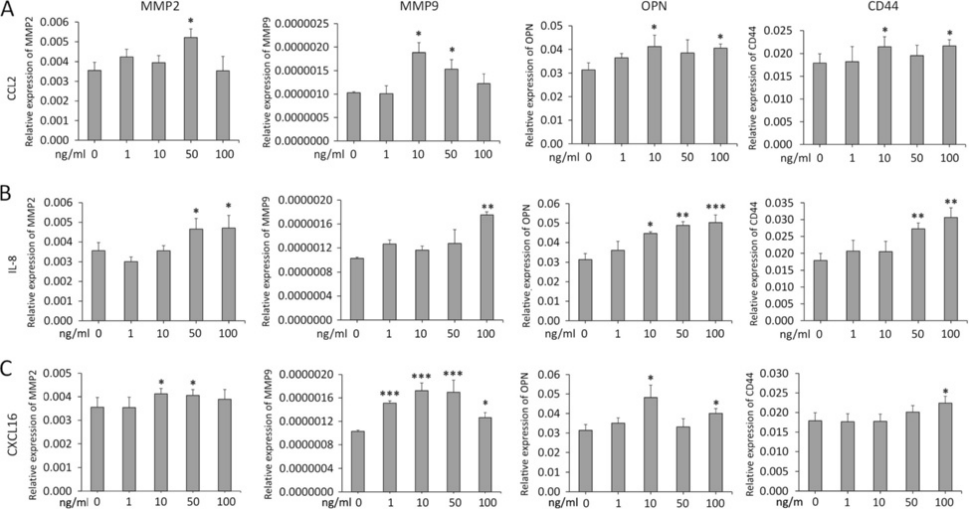
**Regulation of the expression of HCC invasion/metastasis-associated genes by CCL2, IL-8, and CXCL16 in HCC cells.** CCL2 **(A)**, IL-8 **(B)**, and CXCL16 **(C)** induced MMP2, MMP9, OPN, and CD44 expression in MHCC97H cells (*P < 0.05, **P < 0.01, ***P < 0.001 vs. the control).

Three primary cytokines of interest (CCL2, IL-8, and CXCL16) were selected to explore their biological effects on HCC cell invasion and metastasis. The expressions of MMP2, MMP9, OPN, and CD44 genes were upregulated in MHCC97H cells following CCL2, IL-8, or CXCL16 stimulation, but had no obvious dose-dependent effect (Figure 5). It indicated that CCL2, IL-8, and CXCL16 stimulated the high expressions of invasion/metastasis associated genes, and further changed the invasion ability of HCC cells.

### Effects of CCL2, IL-8, or CXCL16 on the activation of the PI3K/Akt, ERK, and NF-κB pathways in HCC cells

**Figure 6:**
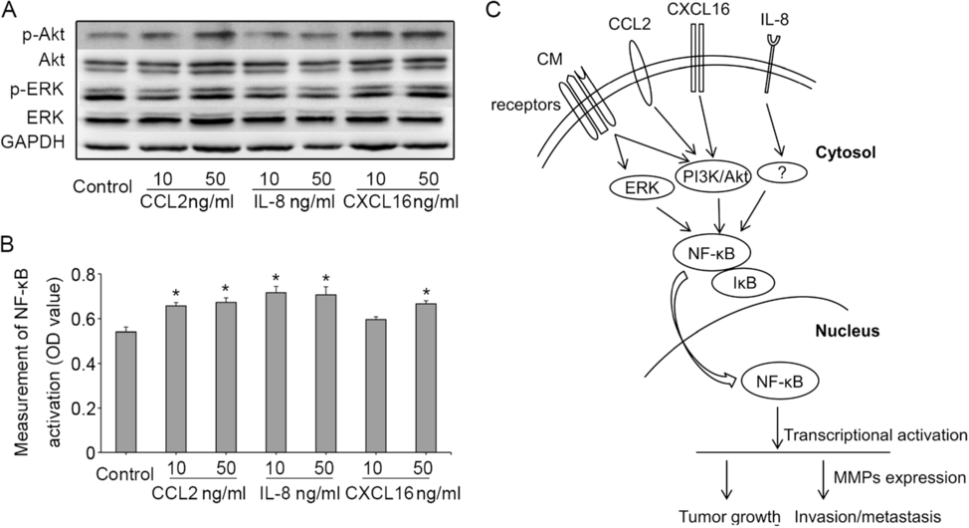
**Effects of CCL2, IL-8, and CXCL16 on the activation of the Akt, ERK, and NF-κB pathways in HCC cells.** The levels of phosphorylated Akt and ERK in MHCC97H **(A)** Cells after exposure to CCL2, IL-8, or CXCL16 at different concentrations. **(B)** Activation of NF-κB in MHCC97H cells were measured using a specific TransAM NF-κB p65 kit under CCL2, IL-8, or CXCL16 stimulation (*P < 0.05 vs. the control). **(C)** Schematic drawing illustrating the proposed mechanism by which endothelial cell-derived cytokines initiated a paracrine signaling cascade that results in enhanced HCC growth and invasion.

As shown in Figure 3, CM increased the activation of the PI3K/Akt and ERK signaling pathways in HCC cells. Accordingly, we next determined whether the differential cytokines CCL2, IL-8, and CXCL16 identified from CM had similar effects on the invasion ability of HCC cells by activating the PI3K/Akt and ERK pathways. After exposure to CCL2 or CXCL16 alone, the AKT phosphorylation level significantly increased in MHCC97H cells, but the ERK phosphorylation level had no change. Additionally, no effects were found on the activation of the Akt and ERK pathways after exposure to IL-8 (Figure 6A). However, the contents of NF-κB all increased compared with that of the control under CCL2, IL-8 or CXCL16 stimulation alone (Figure 6B).

## Discussion

Stroma cells in a tumor microenvironment contribute to the stimulation or modulation of the aggressive behavior of tumor cells. However, to date, the effects of ECs on the malignant biological characteristics of HCC cells are poorly understood. Blood vessel formation and neoangiogenesis are essential to the biological function of ECs. Pro-angiogenic factors secreted from HCC cells such as VEGF, EGF, PDGF, etc. attract various types of ECs from adjacent nontumorous tissues, circulating ECs, or bone marrow-derived endothelial progenitor cells to the site where neoangiogenesis occurs [16]. Meanwhile, ECs isolated from HCC tissue increase the angiogenesis activity with higher resistance to chemotherapeutic agents and inhibitors of angiogenesis [17], and are associated with a high risk for metastasis [18]. In breast cancer, ECs promote tumor cell growth, invasion/metastasis, and the aggressive phenotype [8,19]. In head and neck squamous cell carcinoma, crosstalk initiated by ECs facilitates tumor cell growth, migration, and invasion [9,20]. However, in lung and breast cancers, quiescent HUVEC-conditioned media suppress cell proliferation and invasion [21]. Our study suggested a new paradigm in which EC-initiated signaling directly affects the malignant progression of HCC cells. The HUVECs promoted the tumorigenicity of MHCC97H cells in nude mice and significantly increased the expression of HCC invasion/metastasis-associated genes (MMP2, MMP9, OPN, and CD44). In vitro, CM from HUVECs significantly increased the proliferation of MHCC97H cells, and induced higher expression of MMP2, MMP9, OPN, and CD44 compared with the control medium. Moreover, CM increased the migration and invasion ability of MHCC97H cells (Figures 2C and 2D). These data indicated that HUVECs may participate in regulating tumor growth and invasion through the secreted soluble factors.

Angiogenesis Profiler Array was used here to screen different factors that mediated these effects between tumor cells treated with CM and EBM. A total of 25 differential cytokines were identified, including 22 upregulated and 3 downregulated cytokines in CM. Among them, CCL2, IL-8, and CXCL16 were selected for further biological function exploration based on the following reasons (1) CCL2 was the leading upregulated cytokine in CM but not in EBM. CXCL16 was a moderately upregulated cytokine in CM and had a trace content in EBM. (2) IL8 was a slightly upregulated cytokine in CM but had high contents in CM and EBM. (3) The role of EC-secreted CCL2, IL-8, and CXCL16 in the biological functions of HCC invasion and metastasis is largely unknown.

To clarify the biological effects of CCL2, IL-8, and CXCL16 on HCC cell invasion, we exposed these cells to CCL2, IL-8, and CXCL16 at different concentrations, respectively. The MMP2, MMP9, OPN, and CD44 genes highly expressed in MHCC97H cells under CCL2, IL-8 or CXCL16 stimulation alone like CM stimulation. It indicated that CCL2, IL-8, and CXCL16 stimulation upregulated the expressions of invasion/metastasis associated genes, and further changed the invasion ability of HCC cells. Other studies also favor the significance of cytokine CCL2 in invasiveness and migration of tumor cells such as prostate cancer cells [22,23], breast cancer cells [24] etc. In addition, myofibroblasts-secreted CCL2 also enhances the malignant phenotypes of HCC cells by upregulating MMP2 and MMP9 expression [25], all signs as mentioned above suggest CCL2 involves in pathological development of tumor. However, the secreted CCL2 from ECs influencing HCC cells are little known. CXCL16 and CXCR6 levels increase as tumor malignancy increases in some literatures [26-30]. Soluble CXCL16 chemokine induces proliferation and migration of cancer cells, further regulates invasion and metastasis of cancer [28,30]. In eight hepatoma cells, CXCR6 and its ligand CXCL16 are consistently expressed, and elevated expression of CXCR6 promotes HCC invasiveness and is associated with poor outcomes of patients [31]. These data show CXCL16 stimulation may change the malignant phenotype of HCC cells. The crucial roles of the secreted IL-8 from cancer cells have been validated in tumor growth, angiogenesis, and invasion/metastasis [32-36], and high IL-8 expression is correlated with HCC invasiveness and progression [37,38]. IL-8 can induce the upregulation of MMP7 but has no effects on MMP2 and MMP9 expression in HepG2 cells [39]. On the contrary, in this study, IL-8 stimulation resulted in high expression of MMP2 and MMP9 in MHCC97H cells in a dose-dependent manner (Figure 5B), which might attribute to different malignant phenotypes of MHCC97H and HepG2 cells.

Increased PI3K/Akt and ERK activation reportedly induces the proliferation of HCC cells, prevents HCC cell apoptosis [40], changes the migratory activity and invasiveness of HCC cells [41,42], and is an independent prognostic index for HCC patients [43]. Activation of the PI3K/Akt pathway can enhance MMP2 and MMP-9 expression in HCC and further regulate HCC cell invasion [44,45]. Tumor stromal cells also influence HCC cell invasion ability by activating the PI3K/Akt and ERK pathways [3,25]. In head and neck squamous cell carcinoma, the secreted factors from ECs promote cell migration and invasion by activating the Akt and ERK pathways [9]. A recent study demonstrated that insufficient RFA stimulates EC secretion of IL-6, IL-8, and CCL2 to activate the Akt, ERK, and NF-κB pathways, and further promotes the invasion of HCC cells [15]. Our data suggested that CM from HUVECs enhanced HCC cell migration and invasion, as well as up-regulated HCC invasion/metastasis gene expression in vivo and in vitro. CM also upregulated the phosphorylation levels of Akt and ERK in HCC cells in vivo. These results clearly indicated that CM activating the PI3K/Akt and ERK pathways, as one of the complex signal events, may be involved in the regulation of HCC invasion and metastasis. CCL2, IL-8, and CXCL16, the identified differential cytokines from CM, modulated the expression of HCC invasion/metastasis genes, especially MMP2 and MMP9. CCL2 or CXCL16 alone stimulated significantly the upregulation of phosphorylated AKT in MHCC97H cells, but had no change in ERK phosphorylation. CCL2 or CXCL16 alone also increased the contents of NF-κB compared with the control. These findings hinted that the released CCL2 or CXCL16 from HUVECs may be responsible for HCC cell migration and invasion by increasing MMP2 and MMP9 production through the PI3K/Akt pathway. Other studies on Huh7 cells and chondrosarcoma cells have also revealed a similar molecular mechanism in which CCL2 regulates MMP2 and MMP9 expression through the PI3K/Akt and NF-κB signaling pathways [25,46]. In prostate cancer cells [47], a CXCR6/CXCL16 pair may activate the PI3K/Akt signal pathway. Surprisingly, although IL-8 upregulated the expression of HCC invasion/metastasis genes and increased the contents of NF-κB, it did not affect the activation of the Akt and ERK pathways in MHCC97H. NF-κB is an inducible transcription factor for MMP2 and MMP9 expression in some literatures [46,48,49]. We speculate that IL-8 may activate NF-κB through other signal pathways to regulate the expression of MMP2 and MMP9.

Here, we also mention that the used human cytokine array in the study belongs to a functional protein chip with limited number of cytokine antibodies on it, which is not able to cover all released cytokines from HUVECs. Accordingly, except for 25 identified differential cytokines, the other unidentified cytokines derived from ECs still deserve to be further investigated in the following study.

In summary, several secreted factors from ECs directly influenced HCC cell proliferation, migration, and invasiveness. The differential cytokines CCL2 and CXCL16 identified in CM may be involved in HCC invasion and metastasis by activating the PI3K/Akt and NF-κB signaling pathways. IL-8 may activate NF-κB through other signal pathways to regulate the expression of MMP2 and MMP9 (Figure 6C). Further studies are needed to identify and characterize the signaling events initiated by ECs for future implication in cancer therapy.

## Competing interests

The authors declared that they have no competing interests.

## Authors’ contributions

JFC and ZGR conceived and designed the study, YHW, YYD, WMW, XYX performed the experiments and analyzed the data, YHW and YYD wrote the manuscript. ZMW, RXC contributed statistical analysis. JC, DMG supervised cell and animal experiment. All authors read and approved the final manuscript.
